# Supplementary material for: Inferring interiors and structural history of top-shaped asteroids from external properties of asteroid (101955) Bennu
Source: Nat Commun. 2022 Aug 6;13:4589. doi: 10.1038/s41467-022-32288-y (PMC9357032; doi:10.1038/s41467-022-32288-y)
Supplement: Supplementary file 1 — Supplementary Information [file 41467_2022_32288_MOESM1_ESM.pdf]

SUPPLEMENTARY INFORMATION

Inferring interiors and structural history of top-shaped asteroids from external properties of asteroid (101955) Bennu

Authors: Yun Zhang[1,2], Patrick Michel[1], Olivier S. Barnouin[3], James H. Roberts[3], Michael G. Daly[4], Ronald-L. Ballouz[3,5], Kevin J. Walsh[6], Derek C. Richardson[7], Christine M. Hartzell[2], Dante S. Lauretta[5]

[1]*Université Côte d'Azur, Observatoire de la Côte d'Azur, CNRS, Laboratoire Lagrange, Nice, France.*
[2]*Department of Aerospace Engineering, University of Maryland, College Park, MD, USA.*
[3]*The Johns Hopkins University, Applied Physics Laboratory, Laurel, MD, USA.*
[4]*The Centre for Research in Earth and Space Science, York University, Toronto, ON, Canada.*
[5]*Lunar and Planetary Laboratory, University of Arizona, Tucson, AZ, USA.*
[6]*Southwest Research Institute, Boulder, CO, USA.*
[7]*Department of Astronomy, University of Maryland, College Park, MD, USA.*
**To whom correspondence should be addressed; Email: yun.zhang@oca.eu*

Contents

Supplementary Table

- Supplementary Table 1

Supplementary Figures

- Supplementary Figure 1
- Supplementary Figure 2
- Supplementary Figure 3
- Supplementary Figure 4
- Supplementary Figure 5
- Supplementary Figure 6

Supplementary Table 1: Material properties (ϕ and C), critical spin period ($T_{\rm crit}$), and failure mode under spinup loading of the Bennu-like rubble-pile model with different interparticle parameter sets (μ_S, β, and c).

μ_S	β	c (Pa)	Core density[a]	Structure[b]	ϕ (°)	C (Pa)	$T_{\rm crit}$ (h)[c]	Failure mode[d]
0.2	0.3	0	Underdense	Homogeneous	29	0	3.77	Types I & II[e]
0.2	0.3	0	Uniform	Homogeneous	29	0	3.76	Types I & II
0.2	0.3	0	Denser	Homogeneous	29	0	3.75	Types I & II[f]
0.2	0.3	3	Underdense	Homogeneous	29	0.038	3.77	Types I & II
0.2	0.3	10	Underdense	Homogeneous	29	0.13	3.74	Types I & II
0.2	0.3	30	Underdense	Homogeneous	29	0.38	3.68	Types I & II
0.2	0.3	50	Underdense	Homogeneous	29	0.65	3.62	Types I & II
0.2	0.3	60	Underdense	Homogeneous	29	0.78	3.58	Type II[g]
0.2	0.3	100	Underdense	Homogeneous	29	1.3	3.47	Type II
0.2	0.3	400	Underdense	Homogeneous	29	5.2	2.93	Type IV
0.2	0.3	800	Underdense	Homogeneous	29	10	2.51	Type IV[k]
0.2	0.3	1000	Underdense	Homogeneous	29	13	2.34	Type IV
0.4	0.4	0	Underdense	Homogeneous	32	0	3.53	Types I, II & III[h]
0.4	0.4	0	Uniform	Homogeneous	32	0	3.52	Types I, II & III
0.4	0.4	0	Denser	Homogeneous	32	0	3.52	Types I, II & III
0.6	0.5	0	Underdense	Homogeneous	35	0	3.46	Types I & III[i]
0.6	0.5	0	Uniform	Homogeneous	35	0	3.46	Types I & III
0.6	0.5	0	Denser	Homogeneous	35	0	3.46	Types I & III
0.6	0.5	3	Underdense	Homogeneous	35	0.11	3.44	Types I & III
0.6	0.5	10	Underdense	Homogeneous	35	0.35	3.41	Types I & III
0.6	0.5	50	Underdense	Homogeneous	35	1.7	3.31	Types I & III
0.6	0.5	100	Underdense	Homogeneous	35	3.5	3.02	Types II & III
0.6	0.5	300	Underdense	Homogeneous	35	11	2.50	Type IV
1.0	0.8	0	Underdense	Homogeneous	40	0	3.42	Types I & III[j]
1.0	0.8	0	Uniform	Homogeneous	40	0	3.43	Types I & III
1.0	0.8	0	Denser	Homogeneous	40	0	3.44	Types I & III
1.0	0.8	3	Underdense	Homogeneous	40	0.30	3.40	Types I & III
1.0	0.8	10	Underdense	Homogeneous	40	1.0	3.32	Types I & III

Continuation of Table 1

μ_S	β	c (Pa)	Core density[a]	Structure[b]	ϕ ($^\circ$)	C (Pa)	$T_{\rm crit}$ (h)[c]	Failure mode[d]
1.0	0.8	50	Underdense	Homogeneous	40	5.4	2.88	Types II & III
1.0	0.8	100	Underdense	Homogeneous	40	11	2.57	Type IV
1.0	0.8	200	Underdense	Homogeneous	40	22	2.15	Type IV
0.2	0.3	60	Underdense	Layered	29	0.78	3.58	Types I & II
0.2	0.3	80	Underdense	Layered	29	1.0	3.54	Types I & II[l]
0.2	0.3	100	Underdense	Layered	29	1.3	3.49	Types I, II & III[m]
0.2	0.3	200	Underdense	Layered	29	2.6	3.48	Types I & III[n]
0.2	0.3	400	Underdense	Layered	29	5.2	3.48	Types I & III
0.2	0.3	800	Underdense	Layered	29	10	3.48	Types I & III
0.2	0.3	800	Underdense	Heterogeneous	29	10	3.57	Types I & II
0.2	0.3	8000	Underdense	Heterogeneous	29	100	3.56	Types I & II[o]
0.4	0.4	4300	Underdense	Heterogeneous	32	100	3.35	Types I, II & III
0.6	0.5	2800	Underdense	Heterogeneous	35	100	3.28	Types I & III[p]
1.0	0.8	900	Underdense	Heterogeneous	40	100	3.24	Types I & III

[a] The centre 100-meter-radius region of the rubble-pile model is assigned a density lower than ("Underdense"; $\rho_{\rm inter} = 0.8\rho_{\rm outer}$), equal to ("Uniform"; $\rho_{\rm inter} = \rho_{\rm outer}$), or higher than ("Denser"; $\rho_{\rm inter} = 1.2\rho_{\rm outer}$) the density of the outer region.

[b] All rubble piles use the same μ_S and β to solve the contact interactions for all constituent particles (Methods Section Soft-sphere discrete element method). A homogeneous structure also uses the same c overall for the body, while a layered structure has a 25-meter-depth surface layer with $c = 0$ Pa and a cohesive interior with c given in the third column. A heterogeneous structure has five cohesive regions with the given c in the third column and a coheionless matrix (see Fig. 5a).

[c] The critical spin period is measured at the point when the body's longest axis length has changed by 1.5%. As this pronounced change in the appearance lags behind the structural failure, this value is slightly shorter than the actual critical spin period (ref. [51]).

Continuation of Table 1

μ_S	β	c (Pa)	Core density[a]	Structure[b]	ϕ (°)	C (Pa)	T_{crit} (h)[c]	Failure mode[d]

[d] Type I: local surface landslides, where at least one surface particle having displacement >1 m is detected during spinup; Type II: internal deformation, where changes in the shortest-to-longest axis ratio of the rubble-pile interior exceed 3% (i.e., the 1.5% changes in axis lengths at the end of spinup can be attributed to the deformation of the internal structure; excluding the cases having Type IV failure mode); Type III: surface mass shedding, where at least one surface particle is lofted above the surface (excluding the cases having Type IV failure mode); Type IV: tensile disruption, where surface fracture lineaments are detected during structural reconfiguration (see Supplementary Movies 1–12 for examples of these four failure behaviours).

[e] The structural evolution of this case is presented in Supplementary Movie 1.

[f] See Supplementary Movie 2.

[g] See Supplementary Movie 3.

[h] See Supplementary Movie 4.

[i] See Supplementary Movie 5.

[j] See Supplementary Movie 6.

[k] See Supplementary Movie 7.

[l] See Supplementary Movie 8.

[m] See Supplementary Movie 9.

[n] See Supplementary Movie 10.

[o] See Supplementary Movie 11.

[p] See Supplementary Movie 12.

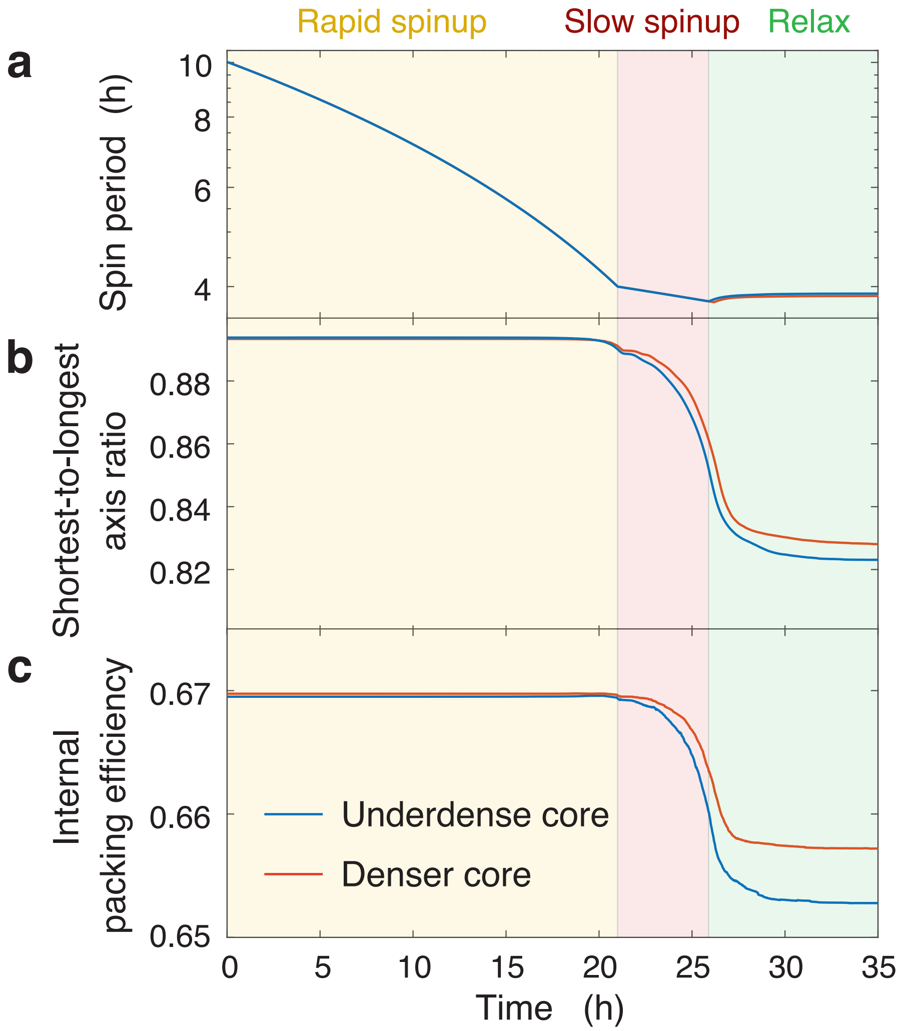

Supplementary Figure 1: **YORP-induced spinup evolution of the Bennu-shaped rubble-pile model with $\phi = 29^{\circ}$ and $C = 0$ Pa.** The rubble pile is forced to rotate following the predefined spin period, including rapid and slow spinup stages (Panel **a**). When the longest axis length has changed by 1.5%, the spinup ceases, and the body evolves under its self-gravity. The spinup process leads to a more oblate shape (Panel **b**) and a lower internal packing efficiency (Panel **c**). Supplementary Movies 1–2 visualise this process. The results for different internal density distributions are denoted in different colours as indicated in the legend. Both cases can generate a more dilute core, supporting the idea that Bennu's underdense core could be formed through such internal deformation processes regardless of the initial density distribution. Source data are provided as a Source Data file.

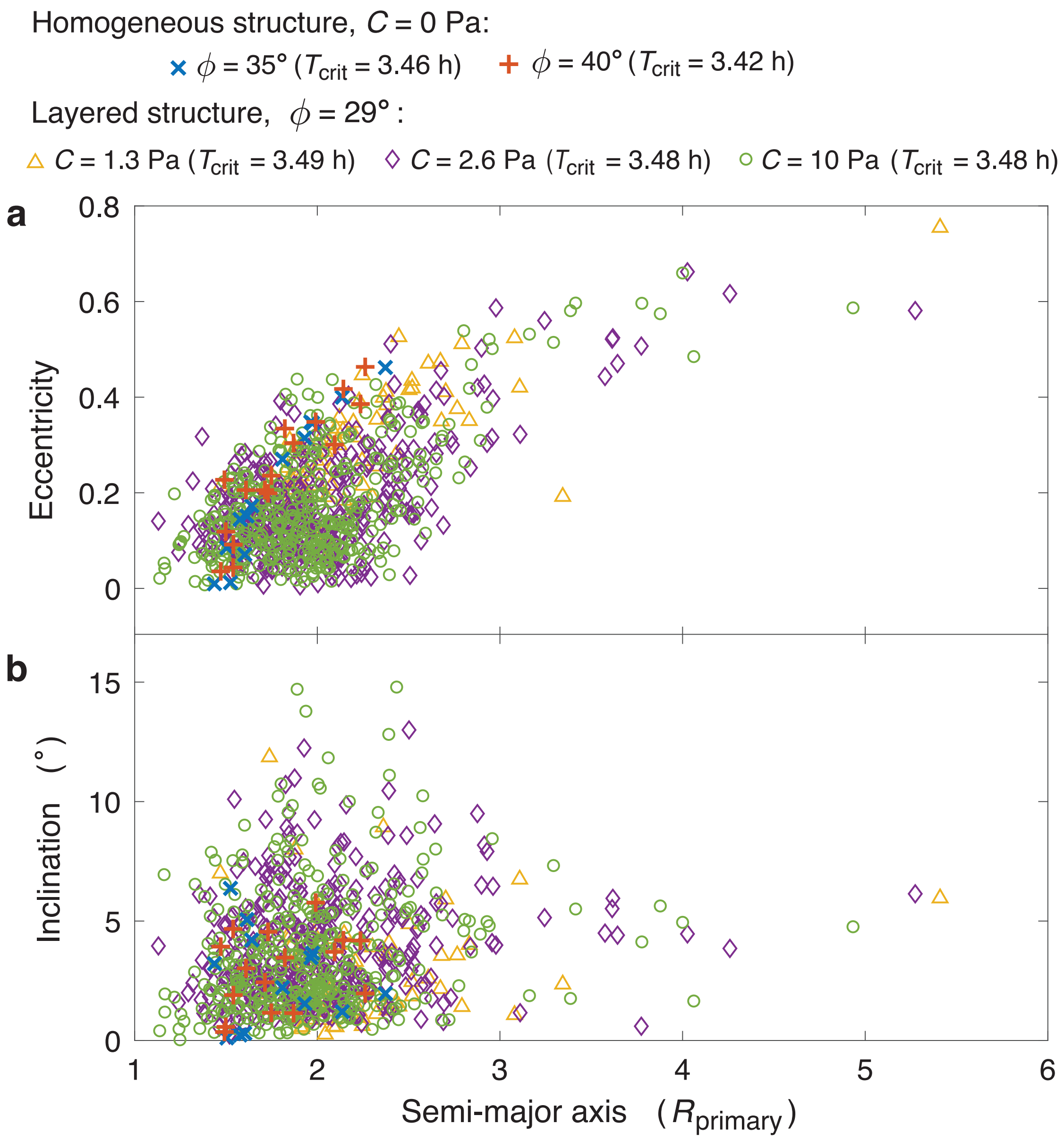

Supplementary Figure 2: **Orbital elements of YORP-induced ejecta around the Bennu-shaped rubble-pile model. a**, orbital eccentricity; **b**, orbital inclination. The semi-major axis is expressed as a ratio relative to the equatorial radius of the primary R_{primary}. The symbols denote the results for tests with different friction angles ϕ and cohesive strength C, as indicated on the top. The results are recorded at the moment 40 h after the initial structural failure for each case. Source data are provided as a Source Data file.

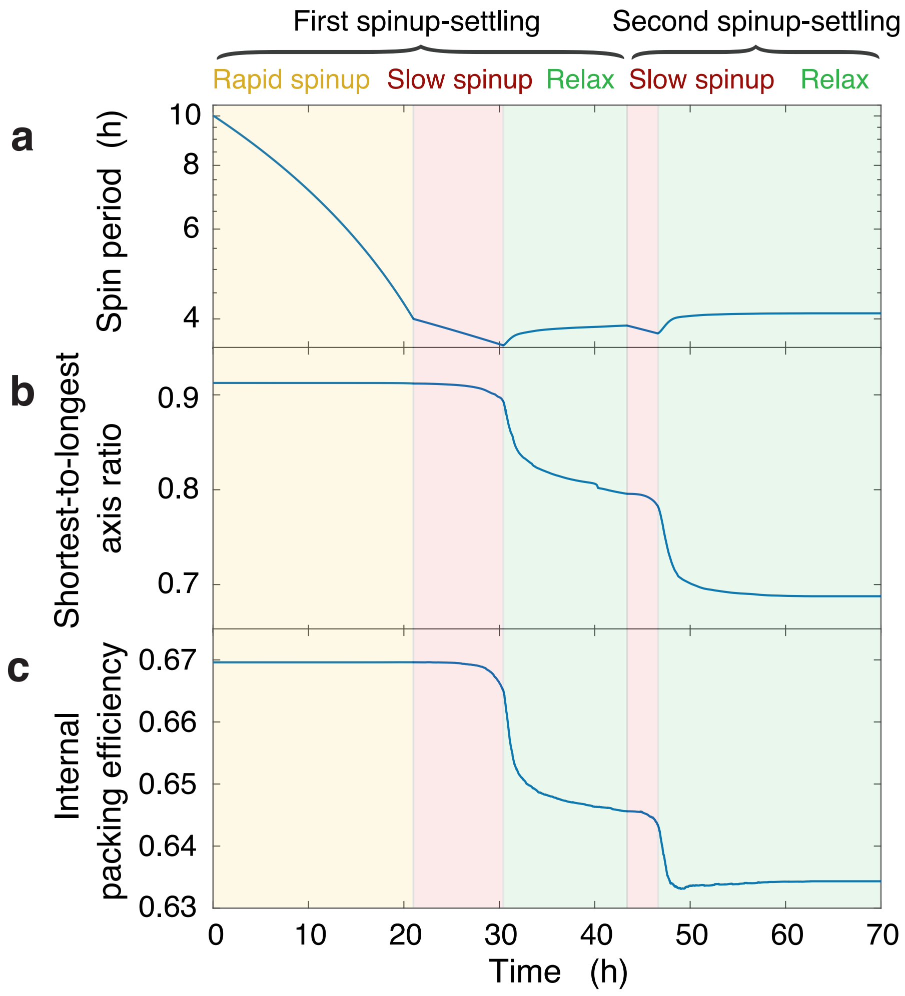

Supplementary Figure 3: **Structural evolution of a heterogeneous rubble pile with $\phi = 29^\circ$ and $C = 0$ Pa undergoing two consecutive spinup-settling paths.** **a**, spin period; **b**, axis ratio; **c**, internal pacing efficiency. During each path, the spinup ceases when the longest axis length has changed by 1.5%, and the body evolves under its self-gravity (see Supplementary Movie 11). The failure behaviours during these two paths are identical, showing that consecutive YORP-spinup cycles could lead a rubble pile to a more oblate shape with a more dilute core. Source data are provided as a Source Data file.

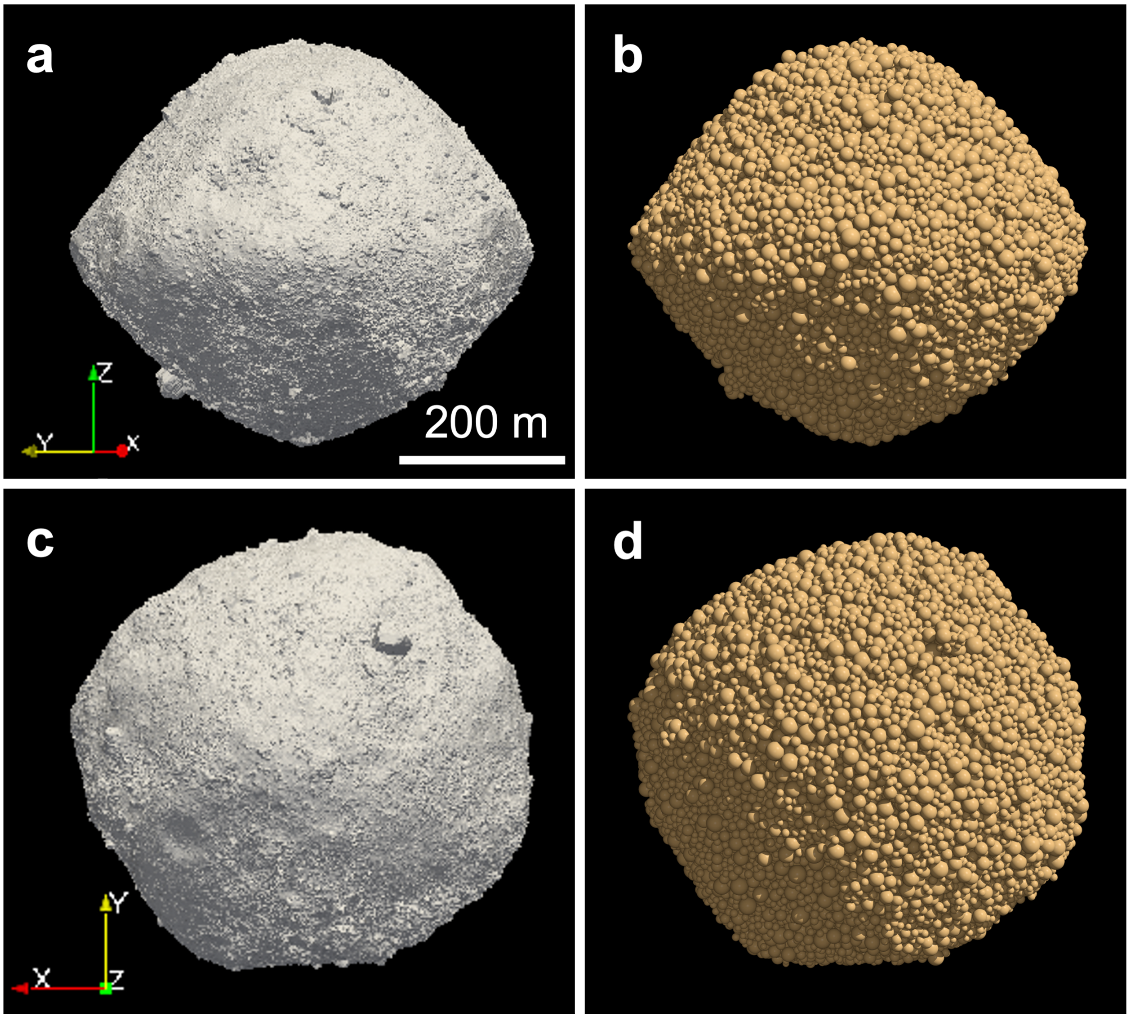

Supplementary Figure 4: **Comparisons of the Bennu OSIRIS-REx Laser Altimeter (OLA) shape model (a, c) and the corresponding rubble-pile model used in this study (b, d).** The top and bottom panels present the top view and side view, respectively. The OLA shape model (v20) is available from the Small Body Mapping Tool (SBMT) at `https://sbmt.jhuapl.edu/Object-Template.php?obj=77`.

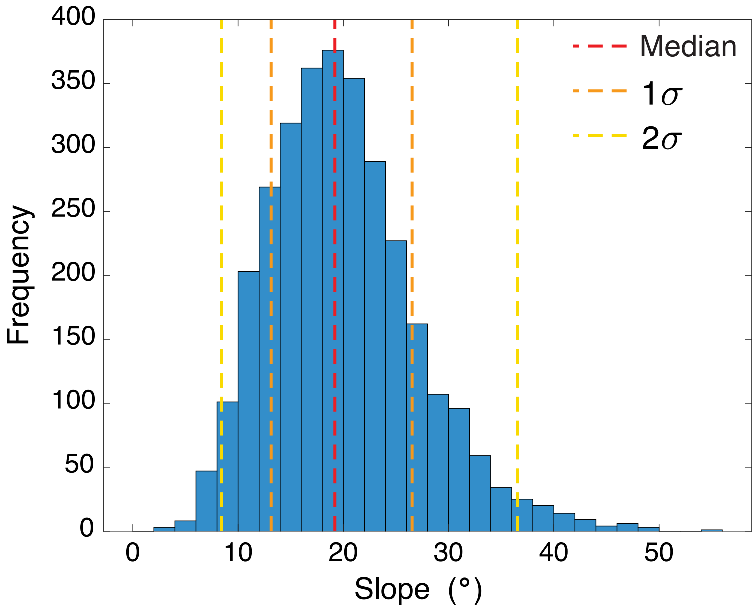

Supplementary Figure 5: **Surface slope distribution of the Bennu-shaped rubble-pile model at Bennu's current spin period of $T = 4.296$ h.** The surface slope is calculated for top-surface particles using Eq. (8) (see Methods). The width of each bin is 2°. Source data are provided as a Source Data file.

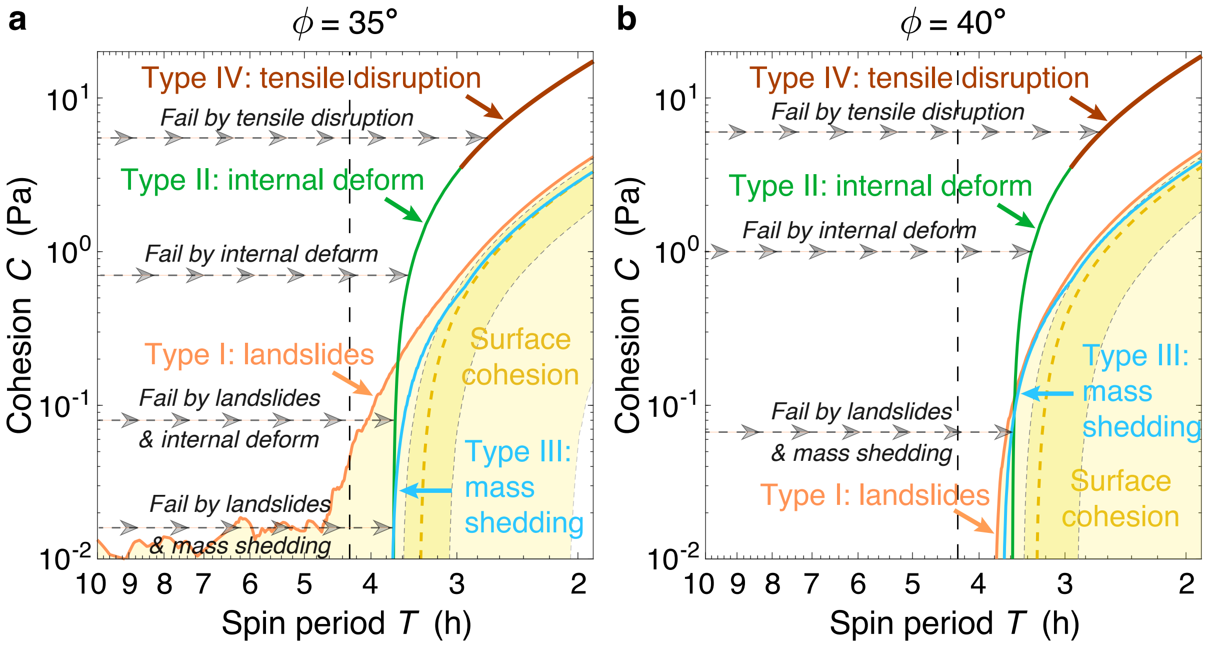

Supplementary Figure 6: **Bennu-shaped cohesive rubble-pile failure mode diagram.** Similar to Fig. 3b, but for $\phi = 35°$ (**a**) and $\phi = 40°$ (**b**). At Bennu's current spin period (as indicated by the black vertical dashed line), surface mass movement is suppressed in the case of $\phi = 35°$ for $C \gtrsim 0.05$ Pa and in the case of $\phi = 40°$ without any cohesion. Source data are provided as a Source Data file.
