# Supplementary material for: Inferring interiors and structural history of top-shaped asteroids from external properties of asteroid (101955) Bennu
Source: Nat Commun. 2022 Aug 6;13:4589. doi: 10.1038/s41467-022-32288-y (PMC9357032; doi:10.1038/s41467-022-32288-y)
Supplement: Supplementary file 3 — Description of Additional Supplementary Files [file 41467_2022_32288_MOESM3_ESM.docx]

**File Name:** Supplementary Movie 1

**Description:** This movie presents the spin-driven structural evolution of a Bennu-shaped rubble pile with a homogeneous structure. The friction angle is 29° and the cohesive strength is 0 Pa (see Supplementary Table 1 for the corresponding discrete element model parameters). The centre 100-meter-radius region of this rubble pile is assigned a density 0.8 times of the density of the outer region. The view is from a non-inertial reference frame corotating with the rubble-pile body. This rubble pile has surface landslides during the spinup process and fails by internal deformation at its spin limit.

**File Name:** Supplementary Movie 2

**Description:** This movie presents the spin-driven structural evolution of a Bennu-shaped rubble pile with a homogeneous structure. The friction angle is 29° and the cohesive strength is 0 Pa (see Supplementary Table 1 for the corresponding discrete element model parameters). This case is similar to that of Supplementary Movie 1, but the centre 100-meter-radius region of this rubble pile is assigned a density 1.2 times of the density of the outer region.

**File Name:** Supplementary Movie 3

**Description:** This movie presents the spin-driven structural evolution of a Bennu-shaped rubble pile with a homogeneous structure. The friction angle is 29° and the cohesive strength is 0.78 Pa (see Supplementary Table 1 for the corresponding discrete element model parameters). The view is from a non-inertial reference frame corotating with the rubble-pile body. This rubble pile does not show any surface landslides during the spinup process and fails by internal deformation at its spin limit.

**File Name:** Supplementary Movie 4

**Description:** This movie presents the spin-driven structural evolution of a Bennu-shaped rubble pile with a homogeneous structure. The friction angle is 32° and the cohesive strength is 0 Pa (see Supplementary Table 1 for the corresponding discrete element model parameters). The view is from a non-inertial reference frame corotating with the rubble-pile body. This rubble pile has surface landslides during the spinup process and fails by internal deformation and mass shedding at its spin limit.

**File Name:** Supplementary Movie 5

**Description:** This movie presents the spin-driven structural evolution of a Bennu-shaped rubble pile with a homogeneous structure. The friction angle is 35° and the cohesive strength is 0 Pa (see Supplementary Table 1 for the corresponding discrete element model parameters). The view is from a non-inertial reference frame corotating with the rubble-pile body. This rubble pile has surface landslides during the spinup process and fails by mass shedding at its spin limit.

**File Name:** Supplementary Movie 6

**Description:** This movie presents the spin-driven structural evolution of a Bennu-shaped rubble pile with a homogeneous structure. The friction angle is 40° and the cohesive strength is 0 Pa (see Supplementary Table 1 for the corresponding discrete element model parameters). The view is from a non-inertial reference frame corotating with the rubble-pile body. This rubble pile has surface landslides during the spinup process and fails by mass shedding at its spin limit.

**File Name:** Supplementary Movie 7

**Description:** This movie presents the spin-driven structural evolution of a Bennu-shaped rubble pile with a homogeneous structure. The friction angle is 29° and the cohesive strength is 10 Pa (see Supplementary Table 1 for the corresponding discrete element model parameters). The view is from a non-inertial reference frame corotating with the rubble-pile body. This rubble pile does not show any surface landslides during the spinup process and fails by tensile disruption at its spin limit.

**File Name:** Supplementary Movie 8

**Description:** This movie presents the spin-driven structural evolution of a Bennu-shaped rubble pile with a layered structure. The friction angle is 29° and the interior cohesive strength is 1.0 Pa (see Supplementary Table 1 for the corresponding discrete element model parameters). The view is from a non-inertial reference frame corotating with the rubble-pile body. This rubble pile has surface landslides during the spinup process and fails by internal deformation at its spin limit.

**File Name:** Supplementary Movie 9

**Description:** This movie presents the spin-driven structural evolution of a Bennu-shaped rubble pile with a layered structure. The friction angle is 29° and the interior cohesive strength is 1.3 Pa (see Supplementary Table 1 for the corresponding discrete element model parameters). The view is from a non-inertial reference frame corotating with the rubble-pile body. This rubble pile has surface landslides during the spinup process and fails by internal deformation and mass shedding at its spin limit.

**File Name:** Supplementary Movie 10

**Description:** This movie presents the spin-driven structural evolution of a Bennu-shaped rubble pile with a layered structure. The friction angle is 29° and the interior cohesive strength is 2.6 Pa (see Supplementary Table 1 for the corresponding discrete element model parameters). The view is from a non-inertial reference frame corotating with the rubble-pile body. This rubble pile has surface landslides during the spinup process and fails by mass shedding at its spin limit.

**File Name:** Supplementary Movie 11

**Description:** This movie presents the spin-driven structural evolution of a Bennu-shaped rubble pile with a heterogeneous structure. The friction angle is 29° and the regional cohesive strength is 100 Pa (see Supplementary Table 1 for the corresponding discrete element model parameters). The view is from a non-inertial reference frame corotating with the rubble-pile body. This rubble pile has surface landslides during the spinup process and fails by internal deformation at its spin limit.

**File Name:** Supplementary Movie 12

**Description:** This movie presents the spin-driven structural evolution of a Bennu-shaped rubble pile with a heterogeneous structure. The friction angle is 35° and the regional cohesive strength is 100 Pa (see Supplementary Table 1 for the corresponding discrete element model parameters). The view is from a non-inertial reference frame corotating with the rubble-pile body. This rubble pile has surface landslides during the spinup process and fails by mass shedding at its spin limit.

**File Name:** Supplementary Information

**Description:** This file includes the supplementary table and figures.

**File Name:** Source Data

**Description:** This file includes the source data for the figures presented in this study.

**File Name:** Supplementary Software 1

**Description:** This file includes two compiled versions of PKDGRAV (one with the spinup module and one without), the user instruction, and three examples for simulating the spin-driven structural evolution presented in this study.
